# Modelling Olfactory Dysfunction in Rodents Through Intranasal Vanadium Exposure

**DOI:** 10.3390/ani16182877

**Published:** 2026-09-12

**Authors:** Margarida Pereira, Sofia Alves-Pimenta, Carlos Venâncio, Maria de Lurdes Pinto, Bruno Colaço

**Affiliations:** 1 School of Agrarian and Veterinary Sciences, University of Trás-os-Montes and Alto Douro (UTAD), Quinta de Prados, 5000-801 Vila Real, Portugal; salves@utad.pt (S.A.-P.); cvenanci@utad.pt (C.V.); lpinto@utad.pt (M.d.L.P.); 2 Centre for the Research and Technology of Agroenvironmental and Biological Sciences (CITAB), Institute for Innovation, Capacity Building and Sustainability of Agri-Food Production (Inov4Agro), University of Trás-os-Montes and Alto Douro (UTAD), Quinta de Prados, 5000-801 Vila Real, Portugal; 3 Veterinary and Animal Science Research Centre (CECAV), Associate Laboratory for Animal and Veterinary Science (AL4AnimalS), University of Trás-os-Montes and Alto Douro (UTAD), Quinta de Prados, 5000-801 Vila Real, Portugal

**Keywords:** olfactory system, olfactory epithelium, olfactory bulb, intranasal delivery, environmental toxicants, neurotoxicity, regeneration 48

## Abstract

Olfactory dysfunction is increasingly recognized as an important consequence of exposure to environmental toxicants, owing to the anatomical and functional characteristics of the olfactory system, which allow inhaled toxicants to reach the central nervous system via the olfactory pathway. Experimental rodent models provide valuable tools for investigating the mechanisms underlying toxicant-induced olfactory injury under controlled conditions. In particular, intranasal vanadium exposure has emerged as a relevant experimental approach because it reproduces an environmentally relevant route of exposure while inducing pathological changes throughout the olfactory system. This review integrates current knowledge derived from experimental rodent models of intranasal vanadium exposure, covering the anatomy and physiology of the olfactory system, intranasal delivery strategies, the cellular and molecular mechanisms underlying vanadium-induced injury, and the associated histopathological, biochemical, behavioural and functional alterations. Vanadium-induced injury is characterized by oxidative stress, mitochondrial dysfunction, neuroinflammation, dysregulation of intracellular signalling pathways, and neuronal degeneration, leading to structural and functional alterations throughout the olfactory pathway. Finally, the review identifies current knowledge gaps regarding olfactory epithelial regeneration and functional recovery following toxic injury and highlights priorities for future research to improve experimental models of environmentally induced olfactory dysfunction.

## Simple Summary

The sense of smell plays an important role in many essential behaviours in animals. The tissues responsible for detecting odours are in direct contact with the external environment and can therefore be damaged by pollutants present in the air. Vanadium, a metal released into the environment from natural sources and human activities, can affect structures involved in olfaction, potentially leading to impaired olfactory function. This review discusses the effects of nasal vanadium exposure on the olfactory system and its use in studying olfactory impairments, bringing together evidence of the changes that occur in the olfactory epithelium and the brain after exposure. Understanding these processes may help improve the use of experimental animal models to investigate the effects of environmental pollutants on the olfactory system.

## 1. Introduction

The olfactory system is essential for mammalian survival and behaviour, enabling the detection and discrimination of environmental chemical cues involved in food acquisition, predator avoidance, reproductive behaviour and social communication [1,2,3]. Unlike other sensory systems, the olfactory system establishes direct contact with the external environment while maintaining direct anatomical connections with the central nervous system (CNS) through the olfactory bulb (OB) [2,3]. Although this unique anatomical organization enables efficient detection of odorants, it also renders the olfactory pathway particularly vulnerable to inhaled environmental pollutants and airborne toxicants, which may access the brain via the olfactory and trigeminal pathways while partially bypassing the blood–brain barrier (BBB) [4,5,6].

From a toxicological perspective, the olfactory epithelium (OE) represents one of the primary interfaces between inhaled xenobiotics and the nervous system [7,8]. Its continuous exposure to airborne contaminants, together with the regenerative capacity of the olfactory neuroepithelium [9,10] and the direct anatomical projection of olfactory sensory neurons (OSNs) to the brain, makes the olfactory system an important experimental model for investigating the neurobiological consequences of inhaled toxicants [4]. Consequently, structural and functional alterations affecting the olfactory pathway are increasingly recognized as sensitive indicators of inhalation-induced neurotoxicity, highlighting the need for experimental models capable of reproducing these pathological processes under controlled conditions [7,11].

Rodents represent a suitable experimental model for studying olfactory dysfunction because their well-characterized olfactory system enables controlled investigation of olfactory function and the pathological mechanisms underlying its dysfunction [1]. Rodent models reproduce many of the cellular and molecular alterations associated with olfactory dysfunction, enabling analysis of the underlying mechanisms and the evaluation of potential therapeutic strategies, making them important tools for translational olfactory research [12,13,14,15]. Moreover, their OE contains the same principal cellular populations found in other mammalian species, including OSNs, sustentacular cells and basal stem cells responsible for lifelong epithelial renewal, while the structural organization of the OB and the mechanisms responsible for odour detection, signal transduction and neuronal regeneration are also conserved [9,10]. Furthermore, rodents rely on olfaction for essential behaviours such as food acquisition, predator avoidance, reproduction, maternal care and social communication, making functional olfactory deficits readily detectable following olfactory injury [1,11]. Finally, the availability of well-established behavioural paradigms, histopathological approaches, genetically modified strains and advanced molecular techniques has established rodents as the predominant experimental model for investigating olfactory physiology, toxicology and regeneration [1,9,13].

Among the environmental toxicants used to develop experimental models of olfactory dysfunction in rodents, vanadium has emerged as one of the most extensively studied agents owing to its widespread environmental occurrence and well-established neurotoxic properties [16]. Environmental and occupational exposure occurs predominantly through inhalation of airborne vanadium-containing particles generated by both natural and anthropogenic sources [13]. Experimental studies have consistently demonstrated that inhaled vanadium induces oxidative stress [17], neuroinflammation [18], mitochondrial dysfunction [19] and neuronal degeneration in several regions of the CNS, including the striatum, substantia nigra [20] and hippocampus [21]. Although research has traditionally focused on its effects in central brain regions, growing evidence indicates that the olfactory system represents one of the earliest and most vulnerable targets of vanadium exposure, with structural and functional alterations affecting both the OE [22] and the OB [23,24].

Given that inhalation is the principal route of environmental exposure to vanadium, intranasal administration has become an important experimental approach for reproducing this route of exposure under controlled laboratory conditions [16]. Intranasal models have emerged as valuable tools for studying the mechanisms underlying toxicant-induced olfactory dysfunction and neurotoxicity [7,13]. By enabling controlled administration of defined doses directly into the nasal cavity, this technique allows investigation of pathological changes in the OE and OB, as well as the possible transport of administered substances to the brain through the olfactory and trigeminal pathways [6,25,26].

This review summarizes current evidence on intranasal vanadium exposure as an experimental model for investigating olfactory dysfunction in rodents. Particular emphasis is placed on the anatomy and physiology of the rodent olfactory system, intranasal delivery approaches, the structural, neurochemical and molecular alterations affecting the OE and OB, behavioural manifestations of olfactory dysfunction, and the cellular and molecular mechanisms underlying vanadium-induced injury. By integrating findings from experimental studies, this review synthesizes current evidence on the pathophysiological mechanisms underlying toxicant-induced olfactory dysfunction, with particular emphasis on epithelial injury, neurodegeneration and tissue regeneration following intranasal vanadium exposure.

## 2. Methods

This narrative review focuses on experimental rodent models of nasal vanadium exposure and their application to the study of olfactory dysfunction. The literature search was performed in the PubMed, Scopus and Web of Science databases between November 2024 and January 2026 using combinations of terms related to the olfactory system (“olfactory system”, “olfactory epithelium”, “olfactory dysfunction”); vanadium exposure (“vanadium”, “vanadate”); nasal administration (“inhalation”, “intranasal”, “intranasal instillation”, “nose-to-brain”); neurotoxicity (“neurotoxicity”, “neurodegeneration”) and experimental rodent models (“animal model”, “rat”, “rats”, “mouse”, “mice”). Additional relevant publications were identified through manual screening of the reference lists of selected articles. Priority was given to experimental studies investigating vanadium exposure by inhalation or intranasal administration in rats and mice, with particular emphasis on structural, molecular and functional alterations within the olfactory system. Studies employing other routes of vanadium administration were included only when they provided complementary evidence that supported the interpretation of findings from nasal exposure models.

## 3. Anatomy and Physiology of the Olfactory System

The olfactory system is essential for rodent survival by detecting and discriminating environmental chemical signals involved in food selection, predator avoidance, social recognition, reproductive and maternal behaviour [1,3]. In addition to odour perception, olfactory information influences emotional processing, memory formation, autonomic regulation and neuroendocrine function through its connections with limbic and hypothalamic structures [3,27].

**Figure 1:**
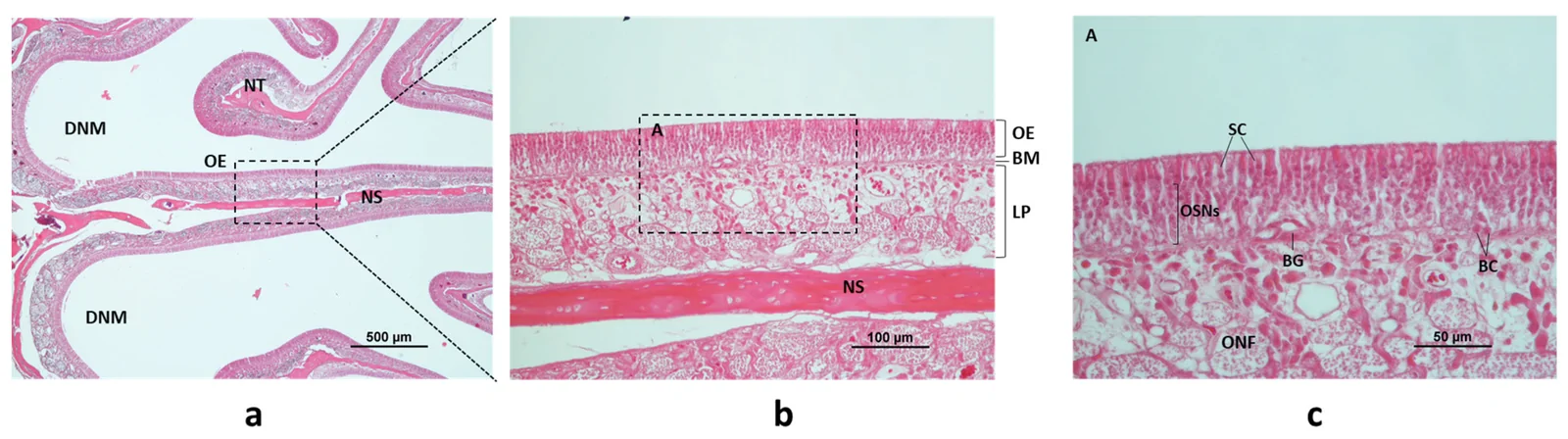
Representative microscopic images showing the histological organization of the rat olfactory epithelium. Nasal cavity tissue was sampled at a standardized anatomical level, paraffin-embedded, sectioned at 3 µm and stained with haematoxylin and eosin (H&E). (**a**) Low-magnification view showing the localization of the olfactory epithelium. (**b**) Higher-magnification view of the boxed region in (**a**), illustrating the organization of the olfactory epithelium and underlying lamina propria. (**c**) High-magnification view of the boxed region (A), showing the principal cellular components of the olfactory epithelium and associated structures within the lamina propria. BC, basal cell; BG, Bowman’s gland; BM, basal membrane; DNM, dorsal nasal meatus; LP, lamina propria; NS, nasal septum; NT, nasal turbinate; OE, olfactory epithelium; ONF, olfactory nerve fascicle; OSNs, olfactory sensory neurons; SC, sustentacular cells. Images obtained by the authors.

The olfactory system consists of peripheral and central components [2,7,28]. The peripheral olfactory system is located within the nasal cavity, where volatile odorants are detected by the OE [7,28]. In rodents, the nasal cavity contains several anatomically and functionally distinct olfactory structures, including the OE, the vomeronasal organ, the septal organ and Grüneberg’s ganglion [28,29,30]. These structures project to specific regions of the main or accessory olfactory bulb, where distinct olfactory inputs are processed through distinct neural pathways [2,30]. The OE occupies the dorsal recess of the nasal cavity, covering the dorsal nasal septum, endoturbinates, ectoturbinates and the region adjacent to the cribriform plate [28,29,30]. The OE is a specialized pseudostratified columnar neuroepithelium composed of OSNs, sustentacular cells, microvillar cells and horizontal and globose basal cells, as illustrated in Figure 1 [9,10].

The OSNs are bipolar neurons whose cell bodies occupy the intermediate layer of the OE [3,28]. Each neuron extends a dendrite towards the epithelial surface, terminating in an olfactory knob from which several non-motile cilia extend into the mucus layer [3,28]. These cilia express odorant receptors and constitute the primary site of olfactory signal transduction [3,31]. From the basal pole of each OSN arises a single unmyelinated axon that crosses the lamina propria, joins adjacent axons to form olfactory nerve fascicles, and passes through the cribriform plate to synapse within the glomeruli of the OB, where the first stage of central olfactory processing takes place [3,28].

Sustentacular cells are essential for maintaining the structural and functional integrity of the OE [3,28]. They contribute to epithelial homeostasis by regulating the ionic and metabolic microenvironment, maintaining the composition of the mucus layer, and providing metabolic support to OSNs [3,7,28]. In addition, they constitute the principal epithelial barrier between the nasal lumen and the underlying nervous tissue and express high levels of xenobiotic-metabolising enzymes, including cytochrome P450 isoforms, flavin-containing monooxygenases and N-acetyltransferases, contributing to the metabolism and detoxification of inhaled xenobiotics and to the antioxidant defence of the OE [32,33]. Bowman’s glands, located within the lamina propria, secrete the serous component of the mucus layer covering the OE, enabling odorant dissolution and delivery to olfactory receptors, contributing to the clearance of odorants, xenobiotics and other inhaled particles from the epithelial surface [7,28,30].

The basal cell population of the OE comprises horizontal basal cells (HBCs) and globose basal cells (GBCs), which are located adjacent to the basal membrane and function as progenitor cells responsible for epithelial maintenance and regeneration [9,34,35]. The OE retains the capacity for lifelong neuronal regeneration [9,10]. GBCs continuously generate new OSNs, whereas HBCs remain quiescent and are activated following epithelial injury [10,35,36]. In rodents, OSNs have an average lifespan of approximately 30–60 days, requiring continuous neuronal replacement to preserve olfactory function [3,37]. Despite continuous neuronal turnover, newly generated OSNs re-establish specific connections with their glomerular targets in the OB, preserving the topographic organization of the olfactory map [9,34,38].

**Figure 2:**
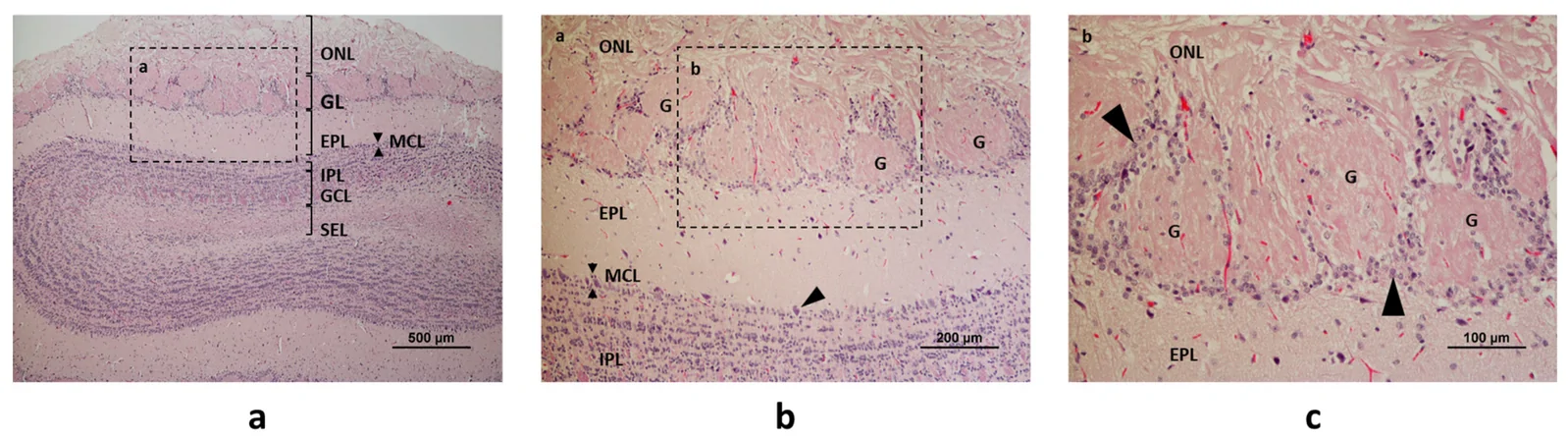
Representative microscopic images showing the histological organization of the rat olfactory bulb. Olfactory bulb tissue was paraffin-embedded, coronally sectioned at 3 µm and stained with haematoxylin and eosin (H&E). (**a**) Low-magnification view showing the laminar organization of the olfactory bulb, including the olfactory nerve layer, glomerular layer, external plexiform layer, mitral cell layer, internal plexiform layer, granule cell layer and subependymal layer. The dashed box indicates the region shown at higher magnification in (**b**). (**b**) Higher-magnification view of the glomerular layer, illustrating the olfactory nerve layer, glomeruli, external plexiform layer and mitral cell layer. Arrowheads indicate mitral cell bodies. The dashed box indicates the region shown at higher magnification in (**c**). (**c**) High-magnification view highlighting the structural organization of the glomeruli, olfactory nerve layer and external plexiform layer. Arrowheads indicate periglomerular cells. EPL, external plexiform layer; G, glomeruli; GCL, granule cell layer; GL, glomerular layer; IPL, internal plexiform layer; MCL, mitral cell layer; ONL, olfactory nerve layer; SEL, subependymal layer. Images obtained by the authors.

The direct anatomical connection between the OE and the CNS is established by the unmyelinated axons of OSNs, which converge into olfactory nerve fascicles that collectively form the olfactory nerve [3,38,39]. The olfactory nerve fascicles traverse the lamina propria and the cribriform plate before terminating within the glomerular layer of the OB [28,38,39]. The OB is a laminated structure composed of the olfactory nerve layer (ONL), glomerular layer (GL), external plexiform layer (EPL), mitral cell layer (MCL), internal plexiform layer (IPL), granule cell layer (GCL), and subependymal layer (SEL) [38,39]. The histological organization of the rat OB is shown in Figure 2.

Within the GL, axons arising from OSNs expressing the same odorant receptor converge onto one or a few glomeruli, allowing each glomerulus to receive sensory input and function as an anatomical and functional unit of olfactory processing [40,41,42,43]. Consequently, odour identity is encoded through the activation of distinct glomerular populations, generating a chemotropic map within the OB [41,42,43]. Within the glomeruli, OSNs establish excitatory synaptic contacts with the dendrites of mitral and tufted cells, the principal projection neurons of the OB, as well as with local interneurons, particularly periglomerular cells, which modulate the processing and integration of olfactory information [41,42,43].

Following initial processing within the OB, mitral and tufted cell axons form the lateral olfactory tract, which projects olfactory information to several forebrain regions, including the anterior olfactory nucleus, piriform cortex, olfactory tubercle, amygdaloid complex and entorhinal cortex [38,43,44]. These projections connect the OB with brain regions involved in odour perception, associative learning, memory, emotional processing, autonomic regulation and motivated behaviours [3,44,45].

Together, the anatomical and functional characteristics make the olfactory system vulnerable to inhaled environmental toxicants [13,45]. This organization allows inhaled substances to interact directly with the olfactory neuroepithelium while also providing a potential route for their transport to the brain through the olfactory pathway [4,5,13]. Consequently, structural or functional alterations affecting any component of the olfactory pathway may impair odour detection, discrimination and processing, rendering the olfactory system particularly susceptible to environmentally induced olfactory dysfunction and neurological disorders [13,45].

## 4. Intranasal Administration: Methods, Advantages and Limitations

Intranasal administration is a well-established route that has been widely applied in both therapeutic and experimental research. Owing to the unique anatomical relationship between the nasal cavity and the CNS, it has become an important strategy for nose-to-brain drug delivery [6,46,47,48]. However, the success of intranasal delivery depends largely on the transport pathways involved and on the administration method employed, both of which influence the deposition, distribution and reproducibility of substance delivery [6,47,49]. Therefore, understanding the mechanisms of nose-to-brain transport, together with the advantages and limitations of the available intranasal approaches, is essential for selecting the most appropriate experimental approach for studies of olfactory dysfunction and neurotoxicity [6,47].

### 4.1. Mechanisms of Nose-to-Brain Transport

Following intranasal administration, compounds may reach the CNS through two principal pathways: an indirect systemic route, involving absorption across the respiratory epithelium followed by entry into the systemic circulation, from which some compounds may cross the BBB [6,25,47,49], and a direct nose-to-brain route mediated by the olfactory and trigeminal pathways [6,50,51]. Transport along the direct nose-to-brain pathway may occur through both intracellular axonal transport and extracellular diffusion via perineural and perivascular channels associated with the olfactory and trigeminal nerves, enabling the movement of compounds from the nasal cavity to the CNS [6,47,49]. The relative contribution of these pathways depends on several factors, including the physicochemical properties of the administered compound, formulation characteristics and the administration technique [6,47]. As the administration technique influences the site of deposition within the nasal cavity, it also determines the accessibility of compounds to the olfactory and respiratory regions. Careful selection of the delivery technique is therefore essential to achieve the proposed experimental outcome [6,47].

### 4.2. Intranasal Delivery Methods: Inhalation vs. Instillation

Experimental nasal exposure in rodents is mainly achieved using two approaches: inhalation and intranasal instillation [6,13,47]. Inhalation exposure delivers aerosolized compounds through normal respiration, resulting in the deposition of particles within the nasal cavity [5,7,48]. This approach reproduces environmental and occupational exposure [52] and is considered a physiologically relevant method for studying airborne toxicants [7,13]. Nasal deposition is influenced by particle diameter, with larger particles tending to deposit in the anterior regions of the nasal cavity, whereas smaller particles are more likely to reach the olfactory region and may also penetrate into the lower respiratory tract. In addition, airflow dynamics, breathing patterns and aerosol characteristics all contribute to the amount of material reaching the olfactory region [5,7,53]. Inhalation studies are also technically demanding because exposure conditions are difficult to standardize, and inhaled compounds may be deposited in the lungs and absorbed systemically, making selective exposure of the olfactory mucosa difficult to achieve [5,13]. Moreover, aerosol generation increases the risk of laboratory environmental contamination and occupational exposure of personnel, requiring specialized inhalation systems and appropriate containment measures [54]. Although technically demanding, inhalation remains the preferred approach for reproducing realistic exposure to airborne pollutants [13].

Intranasal instillation involves the direct administration of a defined volume of solution into the nasal cavity. Several administration techniques have been developed, differing mainly in the depth of administration and the resulting distribution of the instilled solution within the nasal cavity [55,56]. This approach is used in experimental research because it is technically simple and allows precise control of the administered dose and volume, making it suitable for experimental studies of olfactory dysfunction and nose-to-brain transport [6,47,57]. However, the efficiency of intranasal delivery depends on the administration method employed, as well as the administration volume, delivery depth, animal positioning and head orientation, all of which influence intranasal distribution and the efficiency of nose-to-brain transport [55,56,58,59]. Consequently, accurate targeting of the olfactory region remains challenging, and only a small fraction of the administered dose reaches the OE or the CNS under conventional instillation protocols [55,60,61]. Therefore, the effective dose delivered to the target tissue may differ substantially from the nominal administered dose, particularly when different administration techniques or protocols are compared [60,62].

Given the influence of spontaneous breathing on nasal exposure during conventional inhalation, an alternative approach that could be explored in future toxicological studies is artificial inhalation, in which nasal airflow is experimentally controlled [63]. This approach has been used primarily in studies of olfactory physiology, where it enables precise control of inhalation parameters, and its potential application to aerosolized compound delivery has also been proposed [63]. The control of respiratory parameters may be particularly relevant for olfactory exposure, as breathing can influence the amount and distribution of compounds within the olfactory region [61]. By reducing variability associated with spontaneous breathing, artificial inhalation could therefore improve the reproducibility of nasal exposure in future toxicological studies [63].

### 4.3. Intranasal Instillation Techniques

**Figure 3:**
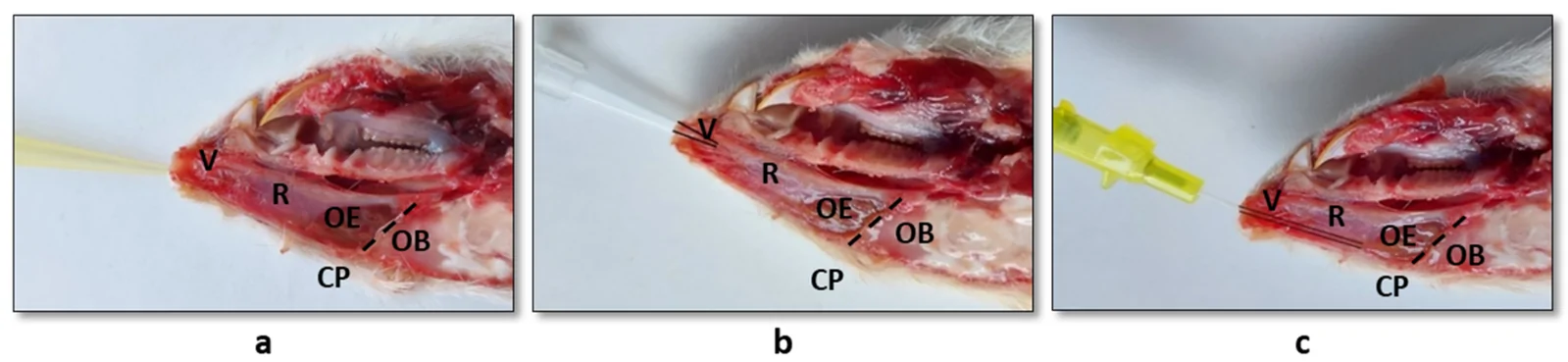
Comparison of the principal intranasal instillation techniques used in experimental rodent models. (**a**) Nose-drop administration, (**b**) administration using a micropipette fitted with a fine tip, and (**c**) cannula-assisted intranasal administration. Representative images were obtained from a sagittal dissected head of a Wistar rat to visualize the nasal cavity and illustrate the relative insertion depth achieved by each technique and its proximity to the olfactory region. OE, olfactory epithelium; OB, olfactory bulb; CP, cribriform plate; R, respiratory epithelium; V, nasal vestibule. Images obtained by the authors.

The principal intranasal instillation techniques used in experimental rodent models are illustrated in Figure 3. These approaches differ in the depth of administration, which determines the distribution of the instilled solution within the nasal cavity and the extent to which the olfactory or respiratory regions are targeted [55,56].

The first approach consists of nose-drop administration, in which the solution is deposited at the entrance of the nostril [55]. This technique does not require specialized equipment or advanced technical skills and is therefore widely used in experimental studies [64]. However, because the administered solution is transported into the nasal cavity by spontaneous inspiration, deposition occurs predominantly within the vestibular and respiratory regions of the nasal cavity, resulting in limited and variable delivery to the olfactory region [55,56,59]. Furthermore, administration of larger volumes may increase drainage into the lower respiratory tract or gastrointestinal tract, thereby reducing deposition within the olfactory region and increasing the risk of pulmonary aspiration [58,64].

A second approach employs a micropipette fitted with a fine tip (nasal tip), which is inserted a few millimetres into the nasal cavity before administration [55,56]. Compared with conventional nose-drop administration, this technique enables deposition in deeper regions of the nasal cavity and may improve delivery to the olfactory region [55,56]. Nevertheless, spatial distribution remains heterogeneous, and consistent targeting of the olfactory region cannot be guaranteed [56,61].

The third approach uses an intranasal catheter, allowing direct administration to the olfactory region [55,56,61]. Among the currently available intranasal instillation techniques for rodents, catheter delivery provides the most consistent deposition within the olfactory region and improves the reproducibility of intranasal delivery compared with more superficial methods [55,56,61]. However, this technique requires anesthesia, technical expertise and careful control of insertion depth to minimize the risk of mucosal injury or incorrect administration [61].

The choice of instillation technique should be guided by the objectives of the experimental study, balancing technical simplicity with the need for accurate targeting of the olfactory region [55,61]. Although catheter-assisted administration currently provides the most reliable approach for delivering compounds to the olfactory region, none of the available instillation techniques achieves selective delivery exclusively to the OE [55,56,61].

### 4.4. Biological Limitations of Intranasal Delivery

The efficacy of intranasal delivery is influenced by the administration technique employed, biological barriers, and the physicochemical properties of the administered compound [26,65]. Mucociliary clearance transports deposited material towards the nasopharynx, reducing the residence time of administered compounds within the nasal cavity, while the mucus layer and local metabolic enzymes may limit epithelial permeation before absorption occurs [26,65]. The physicochemical properties of the administered compound, particularly its molecular weight and lipid solubility, also influence transport across the OE and subsequent nose-to-brain delivery [26,60]. Beyond these biological and physicochemical factors, species-specific anatomical differences should also be considered when selecting experimental animal models and interpreting the outcomes of intranasal delivery studies [7,47]. In rats, the olfactory epithelium occupies approximately 50% of the nasal cavity, representing one of the most extensive olfactory epithelia among commonly used laboratory animals [7,66]. Mice, rabbits and dogs also possess proportionally larger olfactory epithelial regions than primates, consistent with their highly developed olfactory function [8]. Consequently, these anatomical differences influence intranasal deposition patterns and the efficiency of nose-to-brain transport, limiting the direct extrapolation of findings obtained in animal models to humans [6,47].

Therefore, although intranasal administration has become an important route for experimental research, biological barriers, physicochemical properties of the administered compound and interspecies anatomical differences should be carefully considered to maximize delivery efficiency and facilitate the accurate interpretation of findings obtained in animal models [13,47].

## 5. Olfactory Dysfunction

Olfactory dysfunction refers to quantitative and qualitative impairments in olfactory function affecting peripheral and/or central structures of the olfactory system [11,67]. Quantitative disorders include hyposmia, defined as reduced olfactory sensitivity, and anosmia, the complete loss of olfactory function, whereas qualitative disorders include parosmia, characterized by distorted odour perception, and phantosmia, the perception of odours in the absence of an external stimulus [11,67]. Regardless of its clinical presentation, olfactory dysfunction reflects disruption of the structural and/or functional integrity of the olfactory system [11].

Experimental studies have demonstrated that environmental toxicants induce histopathological changes throughout the olfactory system, including degeneration of the OE, loss of OSNs, impaired neurogenesis, disrupted projections to the OB, and secondary pathological changes in central olfactory structures [9,68]. These structural alterations are frequently accompanied by changes in olfactory-guided behaviours in experimental animal models [13,68].

The anatomical organization of the olfactory system provides direct access to the CNS, bypassing the BBB and making it particularly susceptible to inhaled environmental toxicants [5,13,68]. Consequently, the olfactory system has become a valuable experimental model for investigating the mechanisms of environmentally induced neurotoxicity and the progression of neuropathological changes following inhalation exposure [13,68]. Among environmental contaminants, vanadium has been increasingly recognized as a neurotoxic metal because of its capacity to accumulate along the olfactory pathway and induce progressive structural, functional, and behavioural alterations [16,69,70]. Understanding the molecular and cellular mechanisms underlying vanadium-induced neurotoxicity is therefore essential and is discussed in the following section.

### 5.1. Mechanisms of Vanadium-Induced Olfactory Dysfunction

Vanadium is a transition metal whose environmental concentrations have increased as a consequence of industrialization and the widespread combustion of fossil fuels, making inhalation one of the principal routes of exposure for humans and animals [16,69,71]. Vanadium, associated with fine and ultrafine airborne particles, is deposited within the OE following inhalation, from where it may gain access to the CNS through nose-to-brain transport pathways, bypassing the BBB [6,50,70]. Observations in naturally exposed dogs living in polluted urban areas have shown vanadium accumulation throughout the olfactory pathway, with the highest concentrations detected in the OE, followed by the OB and frontal cortex. This distribution was associated with DNA damage, chronic neuroinflammation and neuropathological changes, suggesting that the olfactory route represents an important route for vanadium entry and distribution within the CNS following environmental exposure [72].

Vanadium-induced neurotoxicity results from molecular and cellular mechanisms that compromise the structural and functional integrity of the olfactory system [69,70,71]. These mechanisms comprise oxidative stress, neuroinflammation, mitochondrial dysfunction, disruption of intracellular signalling pathways, and apoptosis, affecting both peripheral and central structures of the olfactory system [70,71].

The toxicity of vanadium is determined by its redox chemistry and structural similarity to phosphate, which disrupts multiple intracellular signalling and metabolic pathways [70,71,73]. Under physiological conditions, vanadium occurs in two oxidation states: pentavalent vanadate (V^5+^), which predominates in the extracellular environment, and tetravalent vanadyl (V^4+^), which predominates intracellularly [71,74,75]. Following cellular uptake, vanadate is reduced to vanadyl by endogenous reducing agents, including glutathione (GSH), ascorbate, nicotinamide adenine dinucleotide phosphate (NADPH), cysteine and catecholamines [74,76,77]. Vanadyl may subsequently be reoxidized to vanadate, establishing a continuous intracellular redox cycle that promotes the generation of reactive oxygen species (ROS), including superoxide anion (O_2_•^−^), hydrogen peroxide (H_2_O_2_) and hydroxyl radicals (•OH), thereby disrupting intracellular redox homeostasis and inducing oxidative stress [73,78,79]. Vanadium also disrupts intracellular iron homeostasis, thereby increasing ROS generation through Fenton-like reactions promoting oxidative damage to lipids, proteins and nucleic acids [80,81]. Its structural similarity to phosphate also enables vanadate to interact with phosphate-dependent enzymes, including protein phosphatases, ATPases and kinases, disrupting phosphorylation-dependent signalling pathways involved in cell survival, proliferation, metabolism and inflammatory responses [70,71,73,82]. Together, these mechanisms promote oxidative stress and disrupt intracellular signalling pathways, contributing to olfactory dysfunction [71,73].

The following section focuses on the cellular and molecular alterations induced by vanadium in the OE and OB, which represent the initial sites of injury following intranasal exposure.

#### 5.1.1. Cellular and Molecular Mechanisms in the Olfactory Epithelium

The OE is the first tissue directly exposed to inhaled vanadium and therefore represents the earliest target of vanadium-induced toxicity [70]. Although oxidative stress is recognised as a central mechanism of vanadium toxicity, direct biochemical evidence of oxidative injury within the OE remains limited [71,74]. The current understanding relies on integrating findings from a limited number of intranasal vanadium studies with mechanistic insights obtained from experimental models of olfactory epithelial injury and regeneration [32,33,83]. Among these complementary models, methimazole-induced olfactory epithelial injury has demonstrated that toxic damage to the OE involves mitochondrial dysfunction and caspase-3-dependent apoptosis of mature OSNs, supporting the concept that oxidative stress-induced mitochondrial injury is a mechanism underlying olfactory epithelial degeneration [84].

Oxidative stress within the OE not only causes direct cellular damage but also initiates the innate immune response required for tissue repair. Injury to OSNs and sustentacular cells leads to the release of danger-associated molecular patterns (DAMPs) and other intracellular mediators, which activate resident epithelial and immune cells through pattern-recognition receptors (PRRs), thereby triggering local inflammatory responses [33,85,86]. This response is characterised by the activation of resident macrophages and the recruitment of additional inflammatory cells, which enable the phagocytic clearance of cellular fragments while releasing cytokines, chemokines and growth factors that promote epithelial repair and regeneration [33,87,88]. Among the principal inflammatory mediators are tumour necrosis factor-α (TNF-α), interleukin-1β (IL-1β) and interleukin-6 (IL-6), which regulate communication between immune cells and olfactory epithelial cells and influence basal stem cell activation, proliferation and differentiation through signalling pathways involved in tissue repair [33,87]. Under normal physiological conditions, this inflammatory response is regulated and temporary. However, when oxidative injury persists, sustained inflammatory signalling can change the function of HBCs, redirecting them from a neuroregenerative programme towards an immune-defensive phenotype, thereby impairing neuronal differentiation [85]. Consequently, epithelial regeneration becomes impaired, limiting the replacement and maturation of newly generated OSNs, compromising the structural and functional integrity of the OE [33,85].

Histopathological evidence of vanadium-induced injury within the nasal cavity was first reported in occupationally exposed workers. Inhalation of vanadium pentoxide was associated with chronic inflammatory alterations of the nasal mucosa, including subepithelial round-cell infiltration within the lamina propria, increased plasma cell infiltration, and vascular hyperaemia [89]. Although these findings were not specific to the OE, these observations provided evidence of chronic occupational exposure to vanadium with inflammatory changes in the nasal mucosa [89].

**Figure 4:**
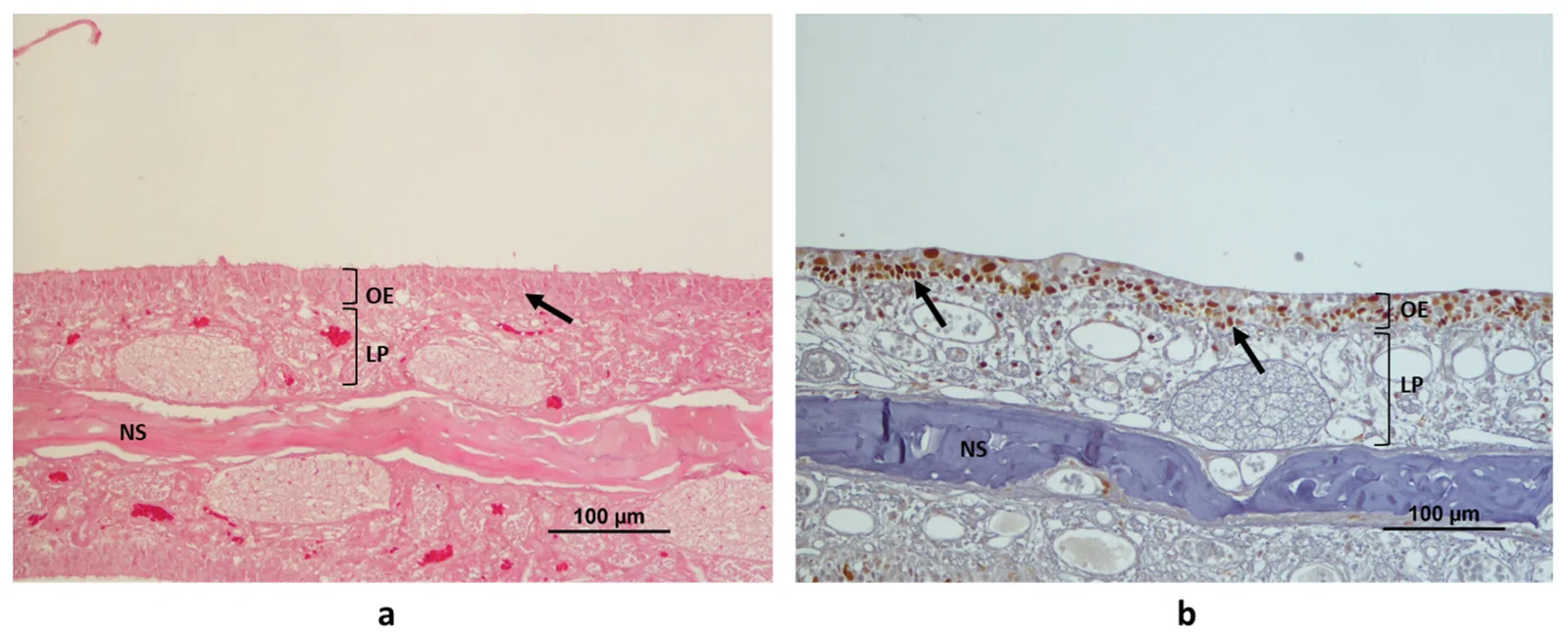
Representative microscopic images showing alterations in the olfactory epithelium of Wistar rats exposed intranasally to 273 µg V_2_O_5_ three times per week for four weeks and euthanized immediately after the last administration. Nasal cavity tissue was sampled at a standardized anatomical level, paraffin-embedded and sectioned at 3 µm. (**a**) Haematoxylin and eosin (H&E)-stained section demonstrating reduced olfactory epithelial thickness and coagulative necrosis (arrow). (**b**) Representative proliferating cell nuclear antigen (PCNA) immunohistochemistry showing brown PCNA-positive nuclei (arrows), consistent with a proliferative response to vanadium-induced injury. NS, nasal septum; OE, olfactory epithelium; LP, lamina propria. Images obtained by the authors.

More recently, an experimental intranasal rodent model provided direct evidence of vanadium-induced injury to the OE. Repeated intranasal administration of vanadium induced epithelial degeneration characterized by coagulative necrosis, sustentacular cell vacuolization, histiocytic infiltration and a marked reduction in OSN density (Figure 4a) [22]. These findings indicate that vanadium may affect multiple cellular populations responsible for maintaining the structural and functional integrity of the OE. The observed histiocytic infiltration may also suggest activation of the innate immune response, supporting the involvement of inflammatory mechanisms in the tissue response to vanadium toxicity [22].

Among these alterations, degeneration of sustentacular cells may represent an important determinant of olfactory epithelial injury. As the principal non-neuronal supporting cells of the OE, sustentacular cells are responsible for xenobiotic metabolism, antioxidant defence, and maintenance of epithelial homeostasis [33,90].

Comparable olfactory epithelial lesions have also been reported in dogs naturally exposed to urban air pollution containing combustion-derived vanadium. These animals exhibited degeneration of OSNs and sustentacular cells and epithelial thinning [72]. Vanadium accumulation was observed along the olfactory pathway, suggesting that it may contribute to the observed lesions [72]. The same study also demonstrated an upregulation of metallothionein I–II, suggesting an adaptive cellular response to metal-induced oxidative stress [72]. Metallothioneins are cysteine-rich metal-binding proteins involved in the regulation of intracellular metal homeostasis and protection against oxidative injury through metal binding and scavenging of ROS [91]. Their increased expression is generally interpreted as an endogenous cytoprotective response against metal-induced oxidative stress [72,91]. Accordingly, Calderón-Garcidueñas et al. [72] suggested that induction of metallothionein I–II reflects activation of an intrinsic defence mechanism aimed at preserving olfactory epithelial integrity despite continuous pollutant exposure. Despite this adaptive response, persistent epithelial degeneration indicates that these endogenous defence mechanisms are insufficient to prevent tissue injury during chronic exposure [72].

Following epithelial injury, the OE retains the capacity to regenerate through the continuous replacement of damaged epithelial cells by basal stem cells [33,92,93]. This regenerative response results from interactions between sustentacular cells, infiltrating immune cells, basal stem cells and locally released cytokines and growth factors [33,92]. Inflammatory mediators released during tissue injury promote the activation of HBCs and proliferation of GBCs, initiating the replacement of OSNs and epithelial cells [33,92,93,94]. Pereira et al. [22] provided evidence of this regenerative response by demonstrating a significant increase in PCNA-positive cells following repeated intranasal administration of vanadium. PCNA immunoreactivity was detected predominantly in basal cells and only occasionally in sustentacular cells and OSNs (Figure 4b). In dogs chronically exposed to urban air pollution, however, only a small number of PCNA-positive basal cells was observed, suggesting limited activation of epithelial regeneration despite persistent epithelial injury [72].

Since the OE is anatomically connected to the OB through the olfactory nerve, pathological changes originating in the OE may extend to the OB, contributing to neuronal degeneration and structural alterations [13].

#### 5.1.2. Cellular and Molecular Mechanisms in the Olfactory Bulb

Experimental studies demonstrate that intranasal vanadium exposure induces structural alterations, including neuronal degeneration [24], neurochemical changes characterized by disruption of dopaminergic neurotransmission [20,23,24], astroglial activation [22,23], and functional deficits manifested as impaired olfactory function [22,23,24], underscoring the particular vulnerability of the OB to vanadium-induced neurotoxicity.

Vanadium exposure promotes lipid peroxidation [95,96,97,98,99,100], as reflected by increased levels of malondialdehyde (MDA) and thiobarbituric acid-reactive substances (TBARS), together with disruption of both enzymatic and non-enzymatic antioxidant defence systems, including superoxide dismutase (SOD), catalase (CAT), glutathione (GSH), glutathione peroxidase (GPx), glutathione S-transferase (GST) and ascorbic acid [24,98,100,101]. These alterations disrupt cellular redox homeostasis, increasing neuronal vulnerability to oxidative injury and contributing to neuronal dysfunction. Although most evidence for these biochemical alterations has been obtained from studies in other brain regions, the available findings contribute to the neuronal injury observed in the OB following intranasal vanadium exposure [20,22,24].

Vanadium-induced oxidative stress also impairs mitochondrial function. Peroxidation of mitochondrial membrane phospholipids increases membrane permeability, allowing cytochrome c to be released into the cytosol [102,103,104]. Once in the cytosol, cytochrome c activates the intrinsic apoptotic pathway through sequential activation of caspase-9 and caspase-3 [102,103]. Activation of this pathway culminates in chromatin condensation, DNA fragmentation and apoptotic neuronal death through the proteolytic cleavage of structural and regulatory proteins [102,105]. Using an in vitro N27 dopaminergic neuronal cell model, Ngwa et al. [103] demonstrated that PKCδ is an important mediator of vanadium-induced apoptosis. Activated caspase-3 mediates the proteolytic cleavage of PKCδ, generating active catalytic fragments that further amplify apoptotic signalling. Evidence supporting the involvement of PKCδ in vanadium neurotoxicity was subsequently provided in vivo. Ngwa et al. [106] reported increased PKCδ expression in the OB of mice following intranasal V_2_O_5_ exposure in a co-exposure model, suggesting that PKCδ signalling contributes to vanadium-induced neuropathological changes within the OB. Besides inducing apoptosis, vanadium modulates several intracellular signalling pathways that contribute to neuroinflammation.

Owing to its close structural similarity to phosphate, vanadate acts as a potent inhibitor of protein tyrosine phosphatases (PTPs), resulting in sustained phosphorylation of multiple intracellular proteins and prolonged activation of signalling pathways, including the mitogen-activated protein kinase (MAPK) family and nuclear factor-kappa B (NF-κB) [107,108,109]. Activation of the stress-responsive JNK and p38 MAPK pathways, together with NF-κB signalling, induces COX-2 expression and activation of Ca^2+^-dependent cytoplasmic phospholipase A_2_ (cPLA_2_) [73,108]. These events enhance arachidonic acid metabolism, increasing the production of prostaglandin E_2_ and pro-inflammatory cytokines, including interleukin-1 (IL-1) and tumour necrosis factor-α (TNF-α) [73,108], thereby amplifying neuroinflammation, reactive glial responses and neuronal injury [73].

**Figure 5:**
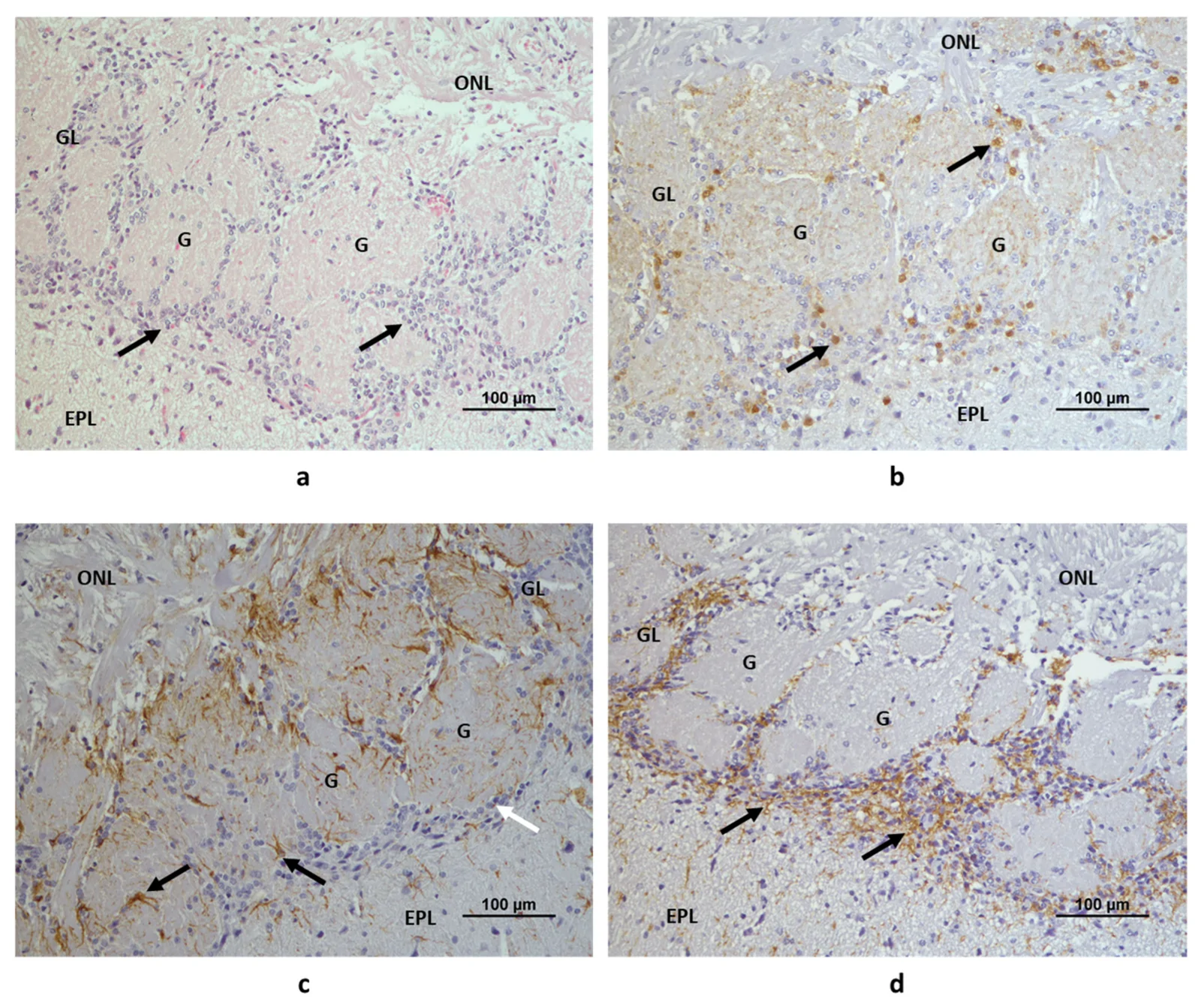
Microscopic images showing histopathological and immunohistochemical alterations in the olfactory bulb of Wistar rats exposed intranasally to 273 µg V_2_O_5_ three times per week for four weeks and euthanized immediately after the last administration. Olfactory bulb tissue was paraffin-embedded, coronally sectioned at 3 µm and processed for histological or immunohistochemical analysis. (**a**) Haematoxylin and eosin (H&E)-stained section showing disruption of the normal histological architecture, including disorganization of the olfactory nerve layer and glomerular layer, with periglomerular cells (black arrows) surrounding the glomeruli. (**b**) Tyrosine hydroxylase (TH) immunohistochemistry showing reduced TH immunoreactivity in the glomerular layer, with brown TH-positive dopaminergic neurons (black arrows) surrounding the glomeruli. (**c**) Glial fibrillary acidic protein (GFAP) immunohistochemistry showing increased GFAP immunoreactivity in the glomerular layer, with brown, star-shaped GFAP-positive astrocytes (black arrows) visible within and surrounding the glomeruli; and periglomerular cells surrounding the glomeruli (white arrow). (**d**) Myelin basic protein (MBP) immunohistochemistry showing brown MBP-immunoreactive fibres in the external plexiform layer and surrounding the glomeruli (black arrows). ONL, olfactory nerve layer; GL, glomerular layer; G, glomerulus; EPL, external plexiform layer. Images obtained by the authors.

The molecular mechanisms described above are associated with the histopathological alterations observed in the OB. Vanadium-induced injury affects multiple structural and cellular components, including afferent olfactory axons, synaptic glomeruli, projection neurons, dendritic spines, myelinated fibres and glial cells, disrupting both structural organization and functional connectivity within the olfactory pathway [20,22,23]. Representative histopathological and immunohistochemical findings in the OB following intranasal vanadium exposure are shown in Figure 5.

Experimental studies have shown neuronal degeneration within the OB following intranasal vanadium exposure [22,24,99]. Histopathological and ultrastructural analyses revealed neuronal shrinkage, chromatin condensation, cytoplasmic vacuolization, mitochondrial swelling, and morphological structures consistent with apoptosis and necrosis, suggesting progressive loss of neuronal integrity [24,99]. These findings are consistent with the cumulative effects of oxidative stress, mitochondrial dysfunction and activation of apoptotic pathways described above. In addition to neuronal degeneration, structural alterations also involve disruption of the ONL, reflecting degeneration of OSN axons projecting from the OE to the OB, and reduction in the number and size of glomeruli [22]. Although the mechanisms underlying degeneration of afferent olfactory fibres have not been directly investigated, oxidative stress and mitochondrial dysfunction may impair axonal integrity, potentially leading to degeneration of these fibres and reduced sensory input to the OB [110].

These alterations may reflect the combined effects of degeneration of afferent olfactory projections and direct vanadium-induced injury within the OB. Experimental models of olfactory epithelial injury have shown that degeneration of OSNs reduces the density of afferent fibres entering the glomeruli, leading to glomerular atrophy and synaptic remodelling [111]. This interpretation is supported by the functional organization of the GL, in which each glomerulus receives convergent input from OSNs expressing the same odorant receptor and forms synaptic connections with mitral and tufted cells, as well as periglomerular interneurons [38]. Consequently, degeneration of afferent olfactory fibres is likely to impair glomerular organization and disrupt the spatial encoding of odour information, thereby compromising central olfactory processing [38,111]. Mitral cell density is also significantly reduced following repeated intranasal vanadium exposure [22]. As the principal projection neurons of the OB, mitral cells constitute the primary efferent pathway to higher olfactory centres [39]. Their loss would be expected to reduce the transmission of olfactory information to higher olfactory centres [111].

In addition to neuronal loss, vanadium exposure also induces a significant reduction in dendritic spine density accompanied by disruption of actin filament organization, indicating impairment of synaptic architecture [24,99]. This structural disruption is accompanied by selective upregulation of matrix metalloproteinase-9 (MMP-9), whereas MMP-2 expression remains unchanged, suggesting activation of extracellular matrix remodelling pathways [112]. Considering the role of MMP-9 in synaptic remodelling and structural plasticity, increased MMP- 9 expression may contribute to dendritic spine loss and neuronal degeneration [112].

Beyond neuronal and dendritic alterations, vanadium also affects myelinated fibres within the OB, resulting in reduced myelin density and disorganization of the ONL [22]. Although the mechanisms underlying these alterations have not been specifically investigated in the OB, experimental studies identify oligodendrocyte progenitor cells as important targets of vanadium toxicity. Vanadium disrupts intracellular iron homeostasis, promoting oxidative stress, mitochondrial dysfunction and apoptosis of oligodendrocyte progenitor cells, thereby impairing oligodendrocyte maturation and normal myelin maintenance [113,114]. In addition, the MMP-9 upregulation described above may contribute to myelin damage through degradation of myelin proteins, including myelin basic protein (MBP) [112]. Consistent with these mechanisms, increased lipid peroxidation together with reduced MBP expression has been associated with myelin sheath disruption following vanadium exposure [97,114].

Alongside neuronal degeneration and myelin disruption, vanadium exposure also increases glial fibrillary acidic protein (GFAP) immunoreactivity, indicating reactive astrogliosis in the OB [22,23,115]. Astrogliosis develops as a secondary response to CNS injury rather than as a direct toxic effect. Neuronal damage triggers the release of extracellular ATP, glutamate, ROS, damage-associated molecular patterns (DAMPs), and pro-inflammatory cytokines, which activate neighbouring astrocytes and initiate the reactive response [116]. Reactive astrocytes show increased GFAP expression, cellular hypertrophy and functional changes that contribute to restoring tissue homeostasis [116].

These structural changes are associated with marked neurochemical alterations within the OB. Vanadium significantly reduces tyrosine hydroxylase (TH) expression [22,23], as well as dopamine (DA) and its principal metabolite, 3,4-dihydroxyphenylacetic acid (DOPAC), suggesting impaired dopaminergic neurotransmission [23]. Dopaminergic interneurons, located mainly within the GL, modulate neurotransmission between OSN terminals and the dendrites of mitral and tufted cells, regulating central olfactory processing [117]. Similar reductions in TH activity, DA and DOPAC levels have been reported following experimental deafferentation of the OB [118], supporting the hypothesis that sensory deafferentation contributes to dopaminergic dysfunction. Vanadium-induced dopaminergic dysfunction may therefore result from both injury to dopaminergic interneurons and secondary alterations in OB circuitry following degeneration of olfactory sensory projections and glomerular remodelling [23].

## 6. Functional Assessment of Olfactory Dysfunction

Although histopathological and neurochemical alterations provide clear evidence of vanadium-induced injury within the olfactory system, behavioural assessment is essential to determine whether these changes result in measurable olfactory deficits. Behavioural assessment complements morphological and molecular analyses by evaluating odour detection, central olfactory processing and odour-guided behaviour, thereby providing an integrated measure of olfactory function [119]. Despite the availability of numerous tests for evaluating olfactory function in rodents, only a limited number have been applied in experimental models of intranasal vanadium exposure. The following paragraphs discuss these behavioural tests and their suitability for assessing vanadium-induced olfactory dysfunction. Table 1 summarises the behavioural assays applied in experimental models of intranasal vanadium exposure, the olfactory domains they evaluate, and the principal findings reported.

The Buried Food Test (BFT) is a commonly used behavioural assay for evaluating odour detection in rodents. It measures the latency for a food-deprived rodent to locate a familiar food item hidden beneath bedding, relying primarily on an intact olfactory system [120,121]. Because it is simple, rapid, and does not require prior training, the BFT is widely used to assess olfactory function in experimental models. Performance in this test may be influenced by locomotor activity, motivation and appetite, factors that should be considered when interpreting the results [120,121].

The social odour investigation test evaluates the spontaneous investigation of socially relevant odours by measuring the time rodents spend sniffing a social stimulus, which serves as an indicator of olfactory function [23]. The test is adapted from the wooden block test [122], which assesses the ability of mice to discriminate between self- and non-self-odours, while incorporating the natural attraction of male rodents to female body odours and urinary pheromones. Reduced investigation time is generally interpreted as indicating impaired odour perception or processing [23].

Unlike behavioural assays based solely on odour detection, the habituation/cross-habituation test assesses rodents’ ability to discriminate between odour stimuli based on their spontaneous exploratory behaviour [123]. Repeated presentation of the same odour progressively reduces sniffing behaviour (habituation). Presentation of a novel odour restores exploratory investigation (cross-habituation or dishabituation), indicating that the animal can discriminate between odour stimuli. Consequently, this test provides a broader assessment of olfactory function than assays only based on odour detection [123]. The neural mechanisms underlying habituation depend on the duration of odour presentation and the intertrial intervals employed, involving processes at the level of the OB or the anterior piriform cortex [123,124]. Under standard behavioural protocols, however, the test primarily assesses spontaneous responses related to odour detection and discrimination [119,123].

Compared with other olfactory behavioural assays, the habituation/cross-habituation test offers several methodological advantages. Because it relies on spontaneous exploratory behaviour, it does not require food or water deprivation, prior training or operant conditioning [123]. This minimizes potential confounding effects related to motivation, metabolic state and learning while allowing repeated assessment of olfactory function over time. By evaluating both odour detection and discrimination, it also provides a more comprehensive evaluation of olfactory function than assays based only on odour detection, such as the BFT [123].

Together, these behavioural assays may provide a more comprehensive characterization of vanadium-induced olfactory dysfunction than any single test alone.

**Table 1:** Experimental rodent models of vanadium exposure by inhalation and instillation and their reported effects on the olfactory system and other regions of the central nervous system (CNS). The table summarizes the exposure method, animal species, vanadium compound, exposure regimen, reported alterations in the olfactory epithelium (OE), olfactory bulb (OB), and other CNS regions, as well as the olfactory function tests and behavioural outcomes used to assess olfactory impairment. ↑ indicates an increase; ↓ indicates a decrease. NR, not reported; TH, tyrosine hydroxylase; DA, dopamine; DOPAC, 3,4-dihydroxyphenylacetic acid; GFAP, glial fibrillary acidic protein; PKCδ, protein kinase C delta; 4-HNE, 4-hydroxynonenal; PCNA, proliferating cell nuclear antigen; ONL, olfactory nerve layer; OSN, olfactory sensory neuron; SOD, superoxide dismutase; GPx, glutathione peroxidase; GR, glutathione reductase; GSH, reduced glutathione; GSSG, oxidized glutathione; GST, glutathione S-transferase; MMP-2, matrix metalloproteinase-2; MMP-9, matrix metalloproteinase-9; SNc, substantia nigra pars compacta; SN, substantia nigra; STR, striatum. * Only the results from animals exposed to vanadium alone are presented.

Exposure Method	Species	Vanadium Compound	Exposure Regimen	OE Alterations	OB Alterations	Other CNS Alterations	Olfactory Function Test	Behavioural Outcome	Reference
Inhalation	Mouse	V_2_O_5_	0.02 M, 1 h, 2×/week	NR	NR	Reduced TH+ neurons in the SNc and dendritic spine loss in the striatum.	NR	-	[20]
Inhalation	Mouse	V_2_O_5_	0.02 M, 1 h, 2×/week	NR	NR	Brain vanadium accumulation and ependymal degeneration (cilia loss, cell sloughing, tight junction disruption).	NR	-	[125]
Inhalation	Mouse	V_2_O_5_	0.02 M, 1 h, 2×/week	NR	NR	Progressive hippocampal degeneration (spine loss, neuronal necrosis and neuropil damage).	NR	-	[21]
Inhalation	Mouse	V_2_O_5_	0.02 M, 1 h, 2×/week	NR	↑ MMP-9 activity; MMP-2 unchanged.	↑ MMP-9 in prefrontal cortex and hippocampus; ↑ MMP-2 in hippocampus and striatum.	NR	-	[112]
Inhalation	Mouse	V_2_O_5_	0.02 M, 1 h, 2×/week	NR	↓ Granule cell size; dendritic spine loss; ultrastructural degeneration; ↑ GPx and GR activity.	NR	Buried Food Test	↑ latency to find the buried food	[24]
Inhalation *	Mouse	V_2_O_5_	0.02 M, 1 h, 2×/week	NR	↓ Granule cell size; dendritic spine loss.	NR	Buried Food Test	↑ latency to find the buried food	[99]
Inhalation	Rat	V_2_O_5_	0.02 M, 1 h, 2×/week	NR	NR	Progressive CA1 neuronal loss with cytoskeletal disruption and neurofibrillary-like changes.	NR	-	[126]
Instillation	Mouse	V_2_O_5_	182 μg V_2_O_5_/50 μL, 3×/week	NR	↓ OB weight; ↓ TH+ neurons; ↓ TH, DA and DOPAC; astroglial activation (↑ GFAP).	Locomotor deficits.	Social discrimination test	↓ female bedding sniffing time	[23]
Instillation	Mouse	V_2_O_5_	97.91 μg V_2_O_5_/50 μL, 3×/week	NR	↓ OB weight; ↓ TH^+^ neurons; ↓ TH, DA and DOPAC; ↑ PKCδ; astrogliosis (↑ GFAP).	↑ Striatal V accumulation; ↑ 4-HNE (STR/SN); ↑ α-synuclein.	Social discrimination test	↓ female bedding sniffing time	[106]
Instillation	Rat	V_2_O_5_	182 μg and 273 μg V_2_O_5_/30 μL, 3×/week	Coagulative necrosis, sustentacular cell vacuolization, histiocytic infiltration, ↓ OSN density; ↑ PCNA-positive basal cells.	Disrupted ONL organization; ↓ glomerular size/number; ↓ mitral cells; gliosis; ↓ TH; focal myelin disruption; ↑ SOD, GSH and GSSG.	Hippocampal gliosis and neuronal death; ↑ GFAP (273 μg); altered GR and GST activity.	Buried Food Test	↑ latency to find the buried food	[22]
							Habituation/cross-habituation test	↓ odour investigation (social/non-social)	
Instillation	Mouse	MnCl_2_/V_2_O_5_ co-exposure	100/75 µg MnCl_2_/V_2_O_5_; 3×/week, 3 months	NR	NR	Brain Mn/V accumulation.	Social discrimination test	↓ time in novel scent zone	[127]

## 7. Experimental Rodent Models of Nasal Vanadium Exposure

Experimental rodent models have become indispensable tools for investigating the neurotoxic effects of vanadium following nasal exposure [20,23]. Unlike systemic administration, nasal exposure reproduces one of the principal environmental and occupational routes through which airborne vanadium-containing particles may enter the body [16]. These models allow simultaneous investigation of local injury to the olfactory mucosa and CNS involvement through the olfactory and trigeminal pathways [4,5,6].

To date, most experimental studies have employed rats or mice exposed to vanadium pentoxide (V_2_O_5_). Despite differences in dose, exposure duration and administration protocols, these models can be divided into four categories according to their primary objective: inhalation models, intranasal instillation models, co-exposure models and neuroprotective intervention models (Table 1).

### 7.1. Inhalation Models

Controlled inhalation models were the first experimental approaches developed to investigate vanadium neurotoxicity and remain the most representative models of environmental and occupational exposure scenarios. In these models, animals are exposed to vanadium-containing aerosols over prolonged periods, closely reproducing chronic inhalation of airborne particulate matter encountered in industrial and urban environments [20,21,24,112]. These studies established inhalation as a biologically relevant route of vanadium neurotoxicity and demonstrated widespread neuropathological alterations involving both the olfactory system and other brain regions [20,21,24]. Because they closely reproduce real-world exposure conditions, inhalation models offer high translational relevance, although the amount of vanadium reaching the OE cannot be precisely controlled.

### 7.2. Intranasal Instillation Models

More recently, repeated intranasal instillation has emerged as an alternative experimental approach that allows precise control of the administered dose while ensuring direct delivery of soluble vanadium compounds to the olfactory mucosa. Compared with inhalation models, intranasal instillation offers greater experimental reproducibility and facilitates the investigation of early pathological events within the olfactory pathway. Consequently, these models have been used to characterize structural alterations of the OE and OB, as well as associated neurochemical, neuroinflammatory and behavioural changes following vanadium exposure [22,23]. This approach is particularly well suited to mechanistic and dose–response studies, owing to the reproducibility of the administration protocol, although it does not fully reproduce the deposition patterns associated with natural inhalation exposure.

### 7.3. Co-Exposure Models

Because environmental and occupational exposures rarely involve a single contaminant, recent studies have incorporated co-exposure paradigms combining vanadium with other airborne neurotoxic metals, particularly manganese [106]. These models aim to reproduce more realistic exposure scenarios and investigate potential interactions between multiple toxicants. Available evidence suggests that combined manganese–vanadium exposure may exacerbate several neurotoxic outcomes compared with exposure to either metal alone. However, these findings should be interpreted cautiously, as the specific contribution of vanadium cannot always be distinguished from that of the co-exposed metal [106].

### 7.4. Models for Testing Neuroprotective Compounds

Some studies have combined vanadium exposure with neuroprotective compounds to investigate whether vanadium-induced neurotoxicity can be prevented or attenuated. These experimental approaches help to elucidate the mechanisms underlying vanadium toxicity while evaluating the neuroprotective potential of the tested compounds [99]. Of the compounds evaluated, carnosine has demonstrated neuroprotective effects by attenuating lipid peroxidation, preserving dendritic spine density, reducing neuronal degeneration, and improving olfactory performance following intranasal vanadium exposure [99]. These findings support the central role of oxidative stress in vanadium-induced neurotoxicity and highlight the potential of these models for identifying and evaluating neuroprotective compounds.

Collectively, the currently available rodent models provide different approaches for studying vanadium-induced neurotoxicity following nasal exposure, each offering distinct advantages depending on the research question. However, relatively few studies have systematically characterized vanadium-induced olfactory dysfunction, as most have focused on isolated structural, molecular or behavioural outcomes. Future studies should integrate structural, molecular and behavioural assessments to provide a more comprehensive understanding of the mechanisms underlying vanadium-induced olfactory dysfunction.

## 8. Conclusions and Future Perspectives

Current evidence demonstrates that nasal vanadium exposure induces structural, neurochemical and functional alterations throughout the olfactory pathway, including degeneration of the OE, disruption of OB organization, dopaminergic dysfunction, astrogliosis and behavioural deficits [22,23,24,112]. These findings establish the olfactory system as a primary target of vanadium neurotoxicity following nasal exposure.

Despite these advances, important knowledge gaps remain. Most experimental studies have focused on characterizing vanadium-induced injury rather than the regenerative processes that follow tissue damage [20,23,24]. The intrinsic regenerative capacity of the olfactory system following vanadium-induced injury therefore remains poorly understood. Although the OE retains the capacity for continuous neuronal regeneration throughout life [91,128], little is known about how vanadium exposure affects the proliferation, differentiation and functional integration of newly generated OSNs. Similarly, the regenerative responses of the OB following vanadium-induced injury remain largely unexplored.

A further limitation of the current literature is the limited integration of structural, molecular and functional assessments within the same experimental model. To our knowledge, only one study of nasal vanadium exposure has simultaneously evaluated structural alterations in the OE and OB together with functional olfactory outcomes [22]. Consequently, the temporal relationship between peripheral epithelial injury, central olfactory alterations and olfactory dysfunction remains poorly understood. Addressing this gap will be essential to clarify how peripheral lesions contribute to central nervous system alterations and ultimately result in olfactory dysfunction following nasal vanadium exposure.

Finally, therapeutic interventions have received limited attention in experimental models of vanadium-induced olfactory dysfunction. Although carnosine has shown neuroprotective effects following intranasal vanadium exposure [99], other approaches with potential neuroprotective or regenerative effects remain largely unexplored in this model. Given the intrinsic regenerative capacity of the olfactory system [92,128], future research should prioritize the development of integrated experimental models combining pathological, molecular and functional assessments of both the OE and the OB, together with therapeutic strategies that not only limit vanadium-induced olfactory injury but also enhance endogenous regenerative processes, promote neuronal repair, facilitate circuit reorganization, and ultimately restore olfactory function. Such advances will improve our understanding of olfactory dysfunction in animals while enhancing the translational relevance of these models for human olfactory dysfunction associated with environmental toxicant exposure.

## Data Availability

No new data were created or analyzed in this study. All information supporting this review is available in the cited literature and the publicly accessible sources referenced in the manuscript.

## Abbreviations

The following abbreviations are used in this manuscript:

BBB	Blood–brain barrier
CNS	Central nervous system
DA	Dopamine
DAMPs	Damage-associated molecular patterns
DMT1	Divalent metal transporter 1
DOPAC	3,4-Dihydroxyphenylacetic acid
GFAP	Glial fibrillary acidic protein
GL	Glomerular layer
IL-1	Interleukin-1
MAPK	Mitogen-activated protein kinase
MMP	Matrix metalloproteinase
MMP-2	Matrix metalloproteinase-2
MMP-9	Matrix metalloproteinase-9
NF-κB	Nuclear factor kappa B
OB	Olfactory bulb
OE	Olfactory epithelium
ONL	Olfactory nerve layer
OSNs	Olfactory sensory neurons
PKCδ	Protein kinase C delta
ROS	Reactive oxygen species
TH	Tyrosine hydroxylase
TNF-α	Tumour necrosis factor
